# Metabolomic profiling for dyslipidemia in pediatric patients with sickle cell disease, on behalf of the IHCC consortium

**DOI:** 10.1007/s11306-022-01954-z

**Published:** 2022-12-02

**Authors:** Hui-Qi Qu, Joseph Glessner, Jingchun Qu, Frank Mentch, Ian Campbell, Patrick Sleiman, John J Connolly, Hakon Hakonarson

**Affiliations:** 1 The Center for Applied Genomics, Children’s Hospital of Philadelphia, 19104 Philadelphia, PA USA; 2 Department of Pediatrics, The Perelman School of Medicine, University of Pennsylvania, 19104 Philadelphia, PA USA; 3 Division of Human Genetics, Children’s Hospital of Philadelphia, 19104 Philadelphia, PA USA; 4 Division of Pulmonary Medicine, Children’s Hospital of Philadelphia, 19104 Philadelphia, PA USA; 5 Faculty of Medicine, University of Iceland, 101 Reykjavik, Iceland

**Keywords:** Dyslipidemia, Free fatty acids, Hypocholesterolemia, Lipoprotein subclasses, Nutrition therapy, Phospholipids, Sickle cell disease

## Abstract

**Background:**

Previous study has shown that dyslipidemia is common in patients with Sickle cell disease (SCD) and is associated with more serious SCD complications.

**Methods:**

This study investigated systematically dyslipidemia in SCD using a state-of-art nuclear magnetic resonance (NMR) metabolomics platform, including 147 pediatric cases with SCD and 1234 controls without SCD. We examined 249 metabolomic biomarkers, including 98 biomarkers for lipoprotein subclasses, 70 biomarkers for relative lipoprotein lipid concentrations, plus biomarkers for fatty acids and phospholipids.

**Results:**

Specific patterns of hypolipoproteinemia and hypocholesterolemia in pediatric SCD were observed in lipoprotein subclasses other than larger VLDL subclasses. Triglycerides are not significantly changed in SCD, except increased relative concentrations in lipoprotein subclasses. Decreased plasma FFAs (including total-FA, SFA, PUFA, Omega-6, and linoleic acid) and decreased plasma phospholipids were observed in SCD.

**Conclusion:**

This study scrutinized, for the first time, lipoprotein subclasses in pediatric patients with SCD, and identified SCD-specific dyslipidemia from altered lipoprotein metabolism. The findings of this study depict a broad panorama of lipid metabolism and nutrition in SCD, suggesting the potential of specific dietary supplementation of the deficient nutrients for the management of SCD.

**Supplementary Information:**

The online version contains supplementary material available at 10.1007/s11306-022-01954-z.

## Introduction

Sickle cell disease (SCD) is a genetic disorder, which is caused by mutation of the hemoglobin subunit beta gene (*HBB*), most commonly seen in African descendants(Diallo, Tchernia 2002; Kato, Piel, Reid, Gaston, Ohene-Frempong, Krishnamurti, Smith et al. 2018). SCD is associated with significant tissue/organ damages and reduced life expectancy(Lubeck, Agodoa, Bhakta, Danese, Pappu, Howard, Gleeson et al. 2019). Previous study has shown that dyslipidemia, abnormally elevated cholesterol or lipids in the blood including hypocholesterolemia and hypertriglyceridemia, is a common comorbid feature of SCD, and is significantly associated with serious SCD complications, e.g. haemolytic severity, vascular dysfunction and pulmonary hypertension.(Akinlade, Adewale, Rahamon, Fasola, Olaniyi, Atere 2014; Zorca, Freeman, Hildesheim, Allen, Remaley, Taylor, Kato 2010) Hypocholesterolemia in SCD includes low levels of total plasma cholesterol and low-density lipoprotein (LDL) cholesterol(El-Hazmi, Jabbar, Warsy 1987; Zorca, Freeman, Hildesheim, Allen, Remaley, Taylor, Kato 2010). However, to date, detailed profiling information as well as the mechanistic basis of dyslipidemia in SCD are lacking. To address this important issue, a metabolomic technology with high reproducibility – the Nightingale nuclear magnetic resonance (NMR) metabolomics platform(Soininen, Kangas, Würtz, Suna, Ala-Korpela 2015; Würtz, Kangas, Soininen, Lawlor, Davey Smith, Ala-Korpela 2017), including 249 biomarkers from 18 metabolite groups, is used in this study to investigate a cohort of SCD cases and controls with the ancestry of African American, recruited at the Center for Applied Genomics (CAG) at the Children’s Hospital of Philadelphia (CHOP). In particular, 98 biomarkers for lipoprotein subclasses, 70 biomarkers for relative lipoprotein lipid concentrations, 18 biomarkers for fatty acids, and 9 biomarkers for phospholipids and other lipids, were investigated by the metabolomics platform.

## Research design and methods

### Subjects

All the pediatric cases and controls were recruited at the CAG center at CHOP between the years of 2002 and 2020, with the ancestry of African American confirmed by the genome-wide SNP genotyping and principal component analysis (PCA) of population structure. Patients with self-identified non-African American ancestry or suggested of non-African American ancestry by PCA were excluded from this study. The upper age limit of the pediatric subjects were 21 years old according to the American Academy of Pediatrics(Hardin, Hackell 2017). No patient had known history of other Mendelian diseases.

### Metabolomic profiling

All the plasma samples were stored at -80℃ until the time of measurement. Samples were processed under a laboratory information management system (LIMS). 249 metabolomic biomarkers for 18 groups were measured using Nightingale nuclear magnetic resonance (NMR) spectroscopy metabolomics platform (Supplementary Table 1)(Nightingale). The large-scale metabolic biomarker profiling platform operates under a strict quality management system. Detailed experimental protocols have been described in the previous publications (Soininen, Kangas, Würtz, Suna, Ala-Korpela 2015; Würtz, Kangas, Soininen, Lawlor, Davey Smith, Ala-Korpela 2017). Biomarker levels (x) were standardized with the mean (µ) and standard deviation (STD, σ), i.e. standardized value = (x – µ)/σ.

### Data analysis

Correlations between SCD and biomarker levels were controlled for age and sex. Statistical analysis was done by the IBM SPSS Statistics Version 23 software. Statistical significance was defined as α = 0.05/249 = 2.01E-04 by correcting for multiple testing of 249 markers.

## Results

**Fig. 1:**
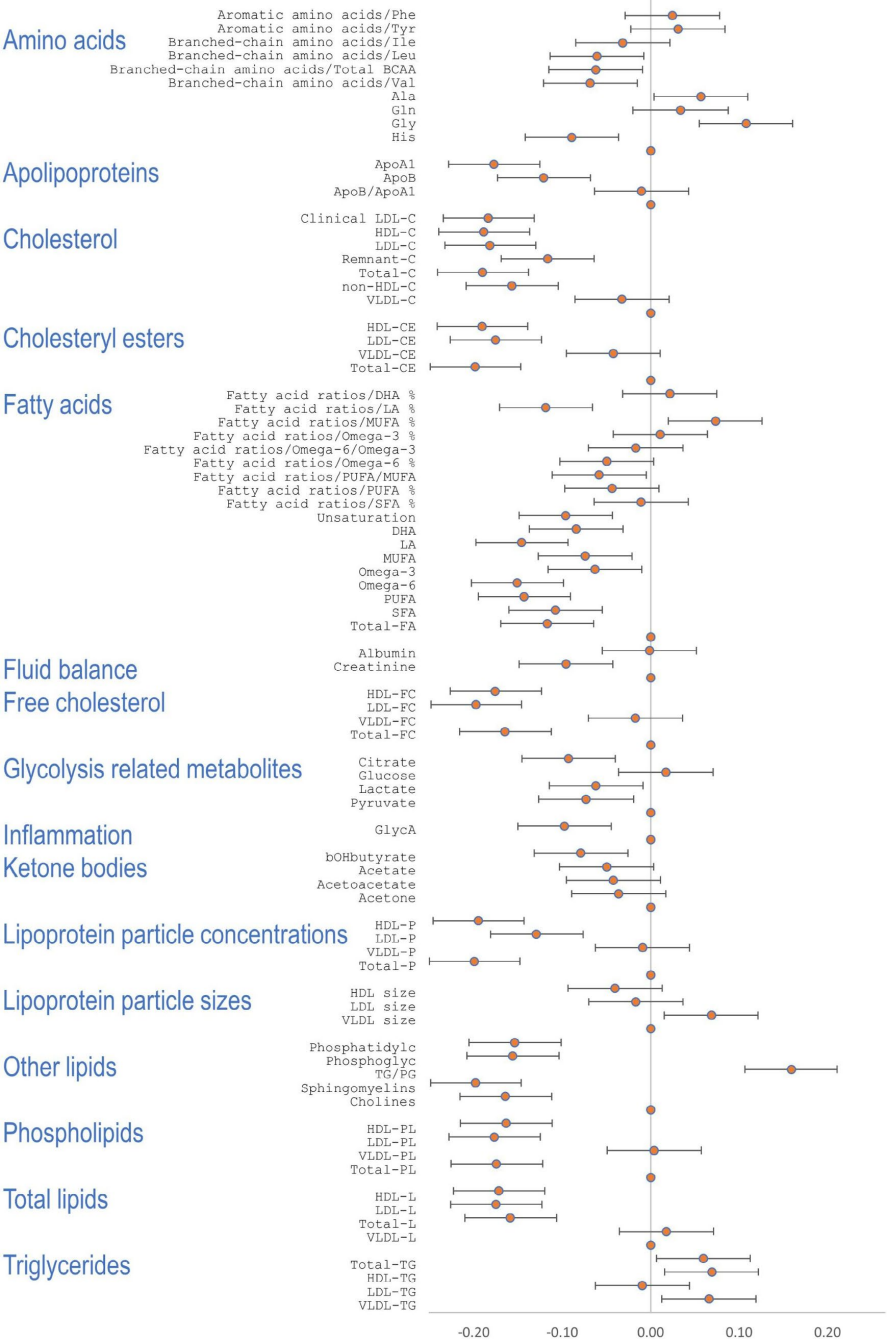
**Forest plot of metabolomic biomarkers.** Correlation coefficient r and 95% confidence intervals are presented as error bars

**Fig. 2:**
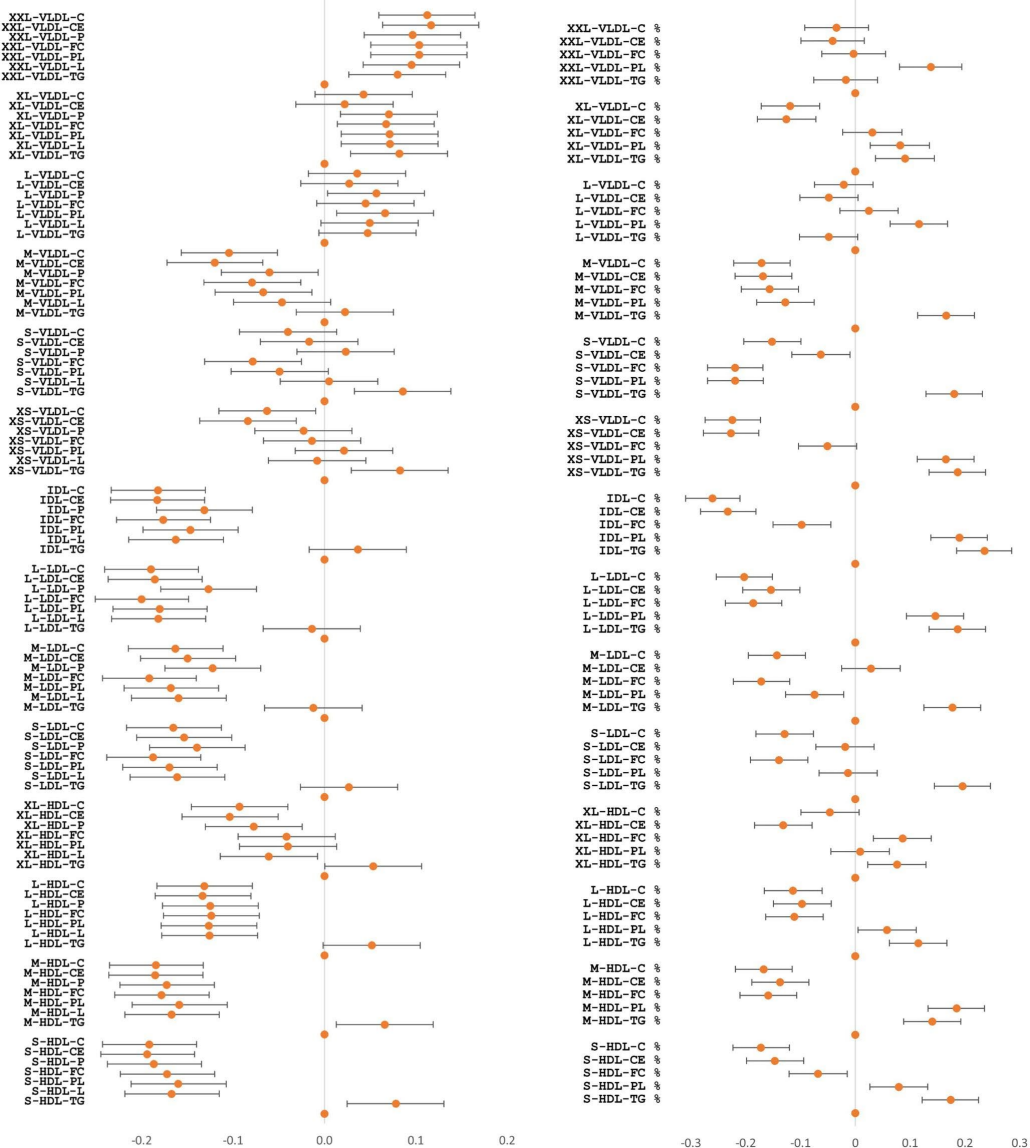
**Forest plot of lipoprotein subclasses and relative lipoprotein lipid concentrations.** Correlation coefficient r and 95% confidence intervals are presented as error bars

This study included 147 cases with SCD (75 males and 72 females, age range 6–21 years old with the average of 15 years), and 1234 controls without SCD (582 males and 652 females, age range 6–21 years old with the average of 15 years). As shown by the correlation between SCD and biomarker levels when controlling for age and sex, 115 biomarkers from 13 groups showed statistical significance (P < 2.01E-04, Table 1; Figs. 1 and 2, Supplementary Table 2). Most significantly, this study observed 41 (41.8%) markers of lipoprotein subclasses (all decreased without exception. Figure 2 left panel) and 38 (54.3%) markers of relative lipoprotein lipid concentrations (Fig. 2 right panel) that were altered in the SCD cases compared to controls, all of which were statistically significance (P < 2.01E-04).

**Table 1:** Number of significant biomarkers correlated with SCD in each metabolomic group

Group	Total	Significant	%
Lipoprotein subclasses	98	41	41.8%
Relative lipoprotein lipid concentrations	70	38	54.3%
Cholesterol	7	6	85.7%
Fatty acids	18	6	33.3%
Other lipids	5	5	100.0%
Cholesteryl esters	4	3	75.0%
Free cholesterol	4	3	75.0%
Lipoprotein particle concentrations	4	3	75.0%
Phospholipids	4	3	75.0%
Total lipids	4	3	75.0%
Apolipoproteins	3	2	66.7%
Amino acids	10	1	10.0%
Inflammation	1	1	100.0%
Fluid balance	2	0	0.0%
Glycolysis related metabolites	4	0	0.0%
Ketone bodies	4	0	0.0%
Lipoprotein particle sizes	3	0	0.0%
Triglycerides	4	0	0.0%
Grand Count	249	115	46.2%

### Total lipids

Except for total lipids in VLDL (VLDL-L), other biomarkers for total lipids are significantly lower in SCD cases, including total lipids in lipoprotein particles (total-L); total lipids in LDL (LDL-L); and total lipids in HDL (HDL-L). Total concentration of lipoprotein particles (total-P), concentration of HDL particles (HDL-P) and concentration of LDL particles (LDL-P), are decreased in SCD cases. Apolipoprotein A1 (ApoA1) and Apolipoprotein B (ApoB) are also decreased in SCD cases.

In lipoprotein subclasses, decreased total lipids include total lipids in IDL (IDL-L), total lipids in large LDL (L-LDL-L), total lipids in medium LDL (M-LDL-L), total lipids in small LDL (S-LDL-L), total lipids in medium HDL (M-HDL-L), and total lipids in small HDL (S-HDL-L). Consistently, decreased concentrations in SCD cases include concentration of IDL particles (IDL-P), concentration of large LDL particles (L-LDL-P), concentration of medium LDL particles (M-LDL-P), concentration of small LDL particles (S-LDL-P), concentration of medium HDL particles (M-HDL-P) and concentration of small HDL particles (S-HDL-P).

### Cholesterol

Significantly decreased cholesterol was observed in SCD cases, in addition to decreased relative concentrations of cholesterol. In contrast, we observed increased relative concentrations of triglycerides and phospholipids in SCD cases. Except for cholesterol, cholesteryl esters (CE), and free cholesterol (FC) in VLDL, cholesterol biomarkers in other classes of lipoproteins are significantly decreased in SCD cases. The decreased markers for plasma total cholesterol, include: total cholesterol (Total-C); LDL cholesterol (LDL-C) and the NMR derived Clinical LDL cholesterol (Clinical LDL-C); total cholesterol minus HDL-C (non-HDL-C); HDL cholesterol (HDL-C); and remnant cholesterol (non-HDL, non-LDL -cholesterol), i.e. Remnant-C. The decreased markers for plasma free cholesterol include total free cholesterol (Total-FC); free cholesterol in LDL (LDL-FC); and free cholesterol in HDL (HDL-FC). The decreased markers for plasma esterified cholesterol include total esterified cholesterol (Total-CE); cholesteryl esters in LDL (LDL-CE); and cholesteryl esters in HDL (HDL-CE).

Hypocholesterolemia by lipoprotein subclasses is also observed and includes:

(1) Decreased total cholesterol in SCD cases, constituting cholesterol in medium VLDL (M-VLDL-C), cholesterol in IDL (IDL-C), cholesterol in large LDL (L-LDL-C), cholesterol in medium LDL (M-LDL-C), cholesterol in small LDL (S-LDL-C), cholesterol in medium HDL (M-HDL-C), and cholesterol in small HDL (S-HDL-C).

(2) Decreased cholesteryl esters in SCD cases, constituting cholesteryl esters in medium VLDL (M-VLDL-CE), cholesteryl esters in very small VLDL (XS-VLDL-CE), cholesteryl esters in IDL (IDL-CE), cholesteryl esters in large LDL (L-LDL-CE), cholesteryl esters in medium LDL (M-LDL-CE), cholesteryl esters in small LDL (S-LDL-CE), cholesteryl esters in medium HDL (M-HDL-CE), and cholesteryl esters in small HDL (S-HDL-CE).

(3) Decreased free cholesterol in SCD cases, constituting free cholesterol in medium VLDL (M-VLDL-FC), free cholesterol in small VLDL (S-VLDL-FC), free cholesterol in IDL (IDL-FC), free cholesterol in large LDL (L-LDL-FC), free cholesterol in medium LDL (M-LDL-FC), free cholesterol in small LDL (S-LDL-FC), free cholesterol in medium HDL (M-HDL-FC), and free cholesterol in small HDL (S-HDL-FC).

Importantly, the changes in cholesterol content by relative lipoprotein lipid concentrations in each lipoprotein subclass were statistically significant for the following categories:

(1) Decreased relative concentrations of total cholesterol in SCD cases, constituting cholesterol to total lipids ratio in IDL (IDL-C %), cholesterol to total lipids ratio in large LDL (L-LDL-C %), cholesterol to total lipids ratio in medium HDL (M-HDL-C %), cholesterol to total lipids ratio in medium LDL (M-LDL-C %), cholesterol to total lipids ratio in medium VLDL (M-VLDL-C %), cholesterol to total lipids ratio in small HDL (S-HDL-C %), and cholesterol to total lipids ratio in small LDL (S-LDL-C %).

(2) Decreased relative concentrations of cholesteryl esters in SCD cases, constituting cholesteryl esters to total lipids ratio in IDL (IDL-CE %), cholesteryl esters to total lipids ratio in large LDL (L-LDL-CE %), cholesteryl esters to total lipids ratio in medium HDL (M-HDL-CE %), cholesteryl esters to total lipids ratio in medium VLDL (M-VLDL-CE %), cholesteryl esters to total lipids ratio in small HDL (S-HDL-CE %), and cholesteryl esters to total lipids ratio in very small VLDL (XS-VLDL-CE %).

(3) Decreased relative concentrations of free cholesterol in SCD cases, constituting free cholesterol to total lipids ratio in large LDL (L-LDL-FC %), free cholesterol to total lipids ratio in medium HDL (M-HDL-FC %), free cholesterol to total lipids ratio in medium LDL (M-LDL-FC %), free cholesterol to total lipids ratio in medium VLDL (M-VLDL-FC %), free cholesterol to total lipids ratio in small LDL (S-LDL-FC %), and free cholesterol to total lipids ratio in small VLDL (S-VLDL-FC %).

Heterogeneity of hypocholesterolemia by lipoprotein classes are highlighted in this study, i.e., decreased cholesterol in SCD cases is significant in IDL, LDL, and HDL, instead of VLDL (Fig. 2). In addition, heterogeneity of VLDL subclasses is observed, i.e., decreased cholesterol in SCD cases is not seen in chylomicrons and extremely large VLDL (XXL-VLDL), very large VLDL (XL-VLDL) or large VLDL (L-VLDL).

### Triglycerides and fatty acids

Overall, triglycerides (TG) were not different between the SCD cases and controls, including total triglycerides (Total-TG), triglycerides in VLDL (VLDL-TG), triglycerides in LDL (LDL-TG), and triglycerides in HDL (HDL-TG). However, relative TG changes are obvious in some lipoprotein subclasses. Increased relative concentrations of triglycerides in SCD cases are consistent with decrease of cholesterol, including triglycerides to total lipids ratio in IDL (IDL-TG %), triglycerides to total lipids ratio in large LDL (L-LDL-TG %), triglycerides to total lipids ratio in medium HDL (M-HDL-TG %), triglycerides to total lipids ratio in medium LDL (M-LDL-TG %), triglycerides to total lipids ratio in medium VLDL (M-VLDL-TG %), triglycerides to total lipids ratio in small HDL (S-HDL-TG %), triglycerides to total lipids ratio in small LDL (S-LDL-TG %), triglycerides to total lipids ratio in small VLDL (S-VLDL-TG %), and triglycerides to total lipids ratio in very small VLDL (XS-VLDL-TG %).

It is also noteworthy that total fatty acids (Total-FA), saturated fatty acids (SFA), polyunsaturated fatty acids (PUFA), omega-6 fatty acids (Omega-6), and linoleic acid (LA), are all significantly lower in SCD cases. In addition, the ratio of linoleic acid to total fatty acids (LA %) is also decreased in SCD cases.

### Phospholipids

Except Phospholipids in VLDL (VLDL-PL), phospholipid biomarkers, including total phospholipids in lipoprotein particles (Total-PL), phospholipids in LDL (LDL-PL), phospholipids in HDL (HDL-PL), are all significantly lower in SCD cases. Decreased phospholipids in SCD cases by lipoprotein subclasses includes phospholipids in IDL (IDL-PL), phospholipids in large LDL (L-LDL-PL), phospholipids in medium LDL (M-LDL-PL), phospholipids in small LDL (S-LDL-PL), phospholipids in medium HDL (M-HDL-PL), and phospholipids in small HDL (S-HDL-PL). In contrast, increased relative concentrations of phospholipids in SCD cases were observed, including phospholipids to total lipids ratio in IDL (IDL-PL %), phospholipids to total lipids ratio in large LDL (L-LDL-PL %), and phospholipids to total lipids ratio in medium HDL (M-HDL-PL %). Additionally, these relative concentrations of phospholipids were observed, although without significance in absolute concentrations, including: (1) Increased relative concentrations in SCD cases: phospholipids to total lipids ratio in chylomicrons and extremely large VLDL (XXL-VLDL-PL %), and phospholipids to total lipids ratio in very small VLDL (XS-VLDL-PL %); (2) Decreased relative concentrations in SCD cases: phospholipids to total lipids ratio in medium VLDL (M-VLDL-PL %), and phospholipids to total lipids ratio in small VLDL (S-VLDL-PL %).

Other phospholipid-related biomarkers, including total cholines, phosphatidylcholines, sphingomyelins, and phosphoglycerides, are significantly lower in SCD cases. Simultaneously, ratio of triglycerides to phosphoglycerides (TG/PG) increased in SCD cases.

### Other biomarkers

This study also observed increased plasma amino acid Glycine (Gly), and decreased inflammation marker glycoprotein acetyls (GlycA) in SCD cases.

## Discussion

### Hypocholesterolemia and relatively increased triglycerides involving lipoprotein subclasses

Considering the important roles of dyslipidemia in serious SCD complications(Akinlade, Adewale, Rahamon, Fasola, Olaniyi, Atere 2014; Zorca, Freeman, Hildesheim, Allen, Remaley, Taylor, Kato 2010), we profiled the plasma lipids in SCD, using the state-of-art Nightingale NMR metabolomics platform. Consistent with previous studies(Akinlade, Adewale, Rahamon, Fasola, Olaniyi, Atere 2014; El-Hazmi, Jabbar, Warsy 1987; Zorca, Freeman, Hildesheim, Allen, Remaley, Taylor, Kato 2010), we observed significantly decreased cholesterol, CE, and FC in SCD cases. Previous study has shown that plasma cholesterol is closely related to hematocrit values, and hypocholesterolemia is also commonly seen in different types of anemia(Westerman 1975). The observed hypocholesterolemia may be due to decreased reservoir storage of cholesterol related to the decreased total red cell mass in SCD anemia(Sasaki, Waterman, Buchanan, Cottam 1983; Westerman 1975). In this study, the decreased cholesterol is mainly seen in IDL, LDL, and HDL.

For the first time, our study shows that decreased cholesterol in SCD cases is not observed in chylomicrons or in extremely large VLDL (XXL-VLDL), very large VLDL (XL-VLDL), or large VLDL (L-VLDL). Normal (or even slightly increased) levels of XXL-VLDL, XL-VLDL, and L-VLDL were observed in this study. In contrast, total triglycerides and triglycerides in each class of lipoprotein showed no significant difference between the SCD cases and controls. Consistently, increased relative concentrations of triglycerides are prominent in lipoprotein subclasses, including M-VLDL, S-VLDL, XS-VLDL, IDL, L-LDL, M-LDL, S-LDL, M-HDL, and S-HDL, in the SCD cases. These findings suggest that metabolic alterations involving triglycerides and total lipids ratios, could have profound effects on the metabolism in children with SCD.

VLDL is produced in the liver from hepatic cholesterol(Tiwari, Siddiqi 2012). Decreased lipoprotein lipase (LPL) activity in SCD has been reported in previous studies(Kim, Jia, Buckett, Liu, Lee, Wessling-Resnick 2013; Vendrame, Olops, Saad, Costa, Fertrin 2019), which may explain relatively increased triglycerides in smaller subclasses of VLDL and IDL, while decreased levels of cholesterol in smaller VLDL suggest decreased lipoprotein levels and reduced rate of lipoprotein metabolism. Decreased LPL activity in SCD may be due to increased levels of endogenous LPL inhibitors, e.g. angiopoietin-like proteins (ANGPTL)(Vendrame, Olops, Saad, Costa, Fertrin 2019), or transfusion iron overload(Kim, Jia, Buckett, Liu, Lee, Wessling-Resnick 2013). As a consequence of lower level of IDL, the production of LDL is decreased.

Decreased total lipids in IDL, L-LDL, M-LDL, S-LDL, M-HDL, and S-HDL in SCD, may be explained by decreased cholesterol as we observed in this study, due to anemia and decreased reservoir storage of cholesterol in SCD(Sasaki, Waterman, Buchanan, Cottam 1983; Westerman 1975; Zorca, Freeman, Hildesheim, Allen, Remaley, Taylor, Kato 2010). Decreased concentrations of IDL particles (IDL-P), large LDL particles (L-LDL-P), medium LDL particles (M-LDL-P), small LDL particles (S-LDL-P), medium HDL particles (M-HDL-P), and small HDL particles (S-HDL-P), can be explained by relatively increased levels of triglycerides, which may be related to the risk factors for high triglycerides, e.g. body weight, diet and physical exercise, as well as hypoxia and inflammatory stress in SCD(Conran, Belcher 2018; Siques, Brito, Leon-Velarde, Barrios, De La Cruz, Lopez, Herruzo 2007; Zorca, Freeman, Hildesheim, Allen, Remaley, Taylor, Kato 2010). In addition, decreased ApoA1 (mainly seen in HDL) and ApoB (mainly seen in VLDL and LDL) can be explained by the hypolipoproteinemia, related to decreased cholesterol and inflammatory stress in SCD(Yalcinkaya, Unal, Oztas 2019).

### Decreased plasma free fatty acids (FFA)

In this study, plasma FFAs total-FA, SFA, PUFA, Omega-6, and linoleic acid, are significantly lower in SCD cases. In addition, LA % is also decreased in SCD cases. With dietary intake as the main source of FFAs, the deficiencies of FFAs may be due to reduced food intake and malnutrition in SCD patients(Prasad, 1997). While hypocholesterolemia is closely related to the severity of anemia in SCD, decreased plasma FFAs may contribute to the various comorbidities observed in SCD. In addition to energy production during fasting, SFA is needed for endogenous cholesterol synthesis(Glatz, Katan 1993). Plasma PUFA is important for brain development and function(Spector 2001), and PUFA deficiency may contribute to negative brain effects observed in SCD patients, in addition to the effects from anemia, decreased oxygen supply, and strokes. Omega-6 has proinflammatory properties(Wendell, Baffi, Holguin 2014). The Omega-6 PUFA linoleic acid as a precursor of arachidonic acid is an essential nutrient for the synthesis of prostaglandins, thromboxanes, and leukotrienes(Mayes, Botham 2003). The decreased levels disclosed by our study highlights the potential of PUFA supplement, especially the Omega-6 PUFA linoleic acid, in the patient care of SCD. Moreover, dietary supplementation of the Omega-3 PUFA α-linoleic acid has been shown of beneficial effects in reducing the pro-inflammatory and pro-thrombotic state in the SCD mouse model(Stivala, Gobbato, Bonetti, Camici, Lüscher, Beer 2022). Therefore, balanced supplementation of Omega-6/Omega-3 warrants for further investigation for its beneficial effects in SCD.

### Phospholipids

Except Phospholipids in VLDL (VLDL-PL), the phospholipid biomarkers total-PL, LDL-PL, and HDL-PL, are all significantly lower in SCD cases. Phospholipids are critical components of the hydrophilic membrane of plasma lipoproteins(Shen et al., 1977). Decreased levels of phospholipid in these lipoproteins, with the hypocholesterolemia described above, may be explained by hypolipoproteinemia in SCD(Vendrame, Olops, Saad, Costa, Fertrin 2018). Interestingly, our study shows that other phospholipid-related biomarkers, including total cholines, phosphatidylcholines, sphingomyelins, and phosphoglycerides, are also significantly lower in SCD cases.

Cholines, phosphatidylcholines, sphingomyelins, and phosphoglycerides, are all essential components of structural lipoproteins(Ueland 2011). In addition, cholines and phosphatidylcholines are precursors of the neurotransmitter acetylcholine(Blusztajn, Liscovitch, Mauron, Richardson, Wurtman 1987). Sphingomyelins play critical roles in cellular signaling(Chakraborty & Jiang, 2013). The decreased levels of cholines, phosphatidylcholines, and sphingomyelins, might thus contribute to cognitive deficits in SCD.

### Other biomarkers

This study also observed increased plasma amino acid Gly, and decreased inflammation marker GlycA in SCD cases. Increased plasma glycine was observed by previous studies(Enwonwu, Xu, Turner 1990; Kiessling, Roberts, Gibson, Ellory 2000), while its biological significance in SCD remains to be elucidated. Decreased GlycA in SCD has been reported before, and the plasma level of GlycA is confounded by hemolysis, thus not a suitable biomarker of inflammation in SCD.(Weisman, Meeks, Mendelsohn, Remaley, Sampson, Allen, Nichols et al. 2018).

In conclusion, this study systematically investigated the observed dyslipidemia in pediatric SCD using a state-of-art NMR metabolomics platform, highlighting for the first time reduced lipoprotein subclasses in SCD hypolipoproteinemia. Specific pattern of hypocholesterolemia in SCD was observed in lipoprotein subclasses, which can’t be recognized by traditional lipid assays. Decreased cholesterol in SCD cases is mainly seen in lipoproteins other than larger VLDL subclasses. On the other hand, triglycerides are not significantly changed in SCD, except increased relative concentrations in lipoprotein subclasses. These changes of cholesterol and triglycerides suggest reduced rate of lipoprotein metabolism. In addition, our study observed decreased plasma FFAs (including total-FA, SFA, PUFA, Omega-6, and linoleic acid) and decreased plasma phospholipids, including cholines, phosphatidylcholines, sphingomyelins, and phosphoglycerides, in patients with SCD. These findings depict a broad panorama of lipid metabolism and nutritional alterations in SCD, suggesting the potential of dietary supplementation of the deficient nutrients for the management of SCD, which warrants further investigation. This study has limitations. As a cross-sectional study, the temporal link between lipoprotein metabolism and SCD could not be determined in this study. A carefully designed longitudinal study based on the CAG biorepository, which has been actively recruiting pediatric patients since 2006, will be able to address this issue. In addition, the mechanistic basis of the SCD-specific dyslipidemia observed in this study warrants for extensive research.

## Electronic supplementary material

Below is the link to the electronic supplementary material.

Supplementary Material 1

Supplementary Material 2

## Data Availability

Supporting data from this study can be obtained by emailing the corresponding author Dr. Hakon Hakonarson.
